# COVID-19 pandemic restrictions continuously impact on physical activity in adults with cystic fibrosis

**DOI:** 10.1371/journal.pone.0257852

**Published:** 2021-09-23

**Authors:** Thomas Radtke, Sarah R. Haile, Holger Dressel, Christian Benden

**Affiliations:** 1 Division of Occupational and Environmental Medicine, Epidemiology, Biostatistics and Prevention Institute, University of Zurich and University Hospital Zurich, Zurich, Switzerland; 2 Division of Chronic Disease Epidemiology, Epidemiology, Biostatistics and Prevention Institute, University of Zurich, Zurich, Switzerland; 3 Faculty of Medicine, University of Zurich, Zurich, Switzerland; Istanbul University Istanbul Faculty of Medicine: Istanbul Universitesi Istanbul Tip Fakultesi, TURKEY

## Abstract

**Background:**

We have recently reported reduced physical activity (PA) in people with cystic fibrosis (pwCF) with and without lung transplantation (LTX) during a 6-week stringent lockdown in Switzerland. This follow-up study explores the impact of coronavirus-2019 disease (COVID-19) related pandemic restrictions on individuals’ therapy regimens and health-related aspects in pwCF.

**Methods:**

We conducted a cross-sectional web-based national survey in Spring 2021. The survey included questions on daily PA, airway clearance and inhalation therapy, questions on COVID-19-compatible symptoms, diagnostic tests and vaccination status, and enquired health-related aspects covering the pandemic period between March 2020 to April 2021.

**Results:**

193 individuals with CF (53% female; 25% LTX recipients) participated. Among pwCF, 10 reported COVID-19 (n = 2 LTX recipients), two subjects were hospitalized, no invasive ventilation required, no deaths. The clinical course was generally mild. Overall, 46% reported less PA during the pandemic, mostly due to closed fitness facilities (85%), lack of motivation (34%), and changes in daily structures (21%). In contrast, 32/193 (17%) pwCF were able to increase their PA levels: 12 (38%) and 11 (34%) reported undertaking home-based training and outdoor activities more frequently; 6 (19%) reported an increase in routine PA, and another 3 (9%) started new activities. Among pwCF without LTX, 5% and 4% reported to undertake less airway clearance and inhalation therapy, respectively.

**Conclusions:**

Our study reveals unfavorable consequences of COVID-19 pandemic restrictions on PA of pwCF with unknown long-term consequences for their overall physical fitness and lung health. Strategies to overcome this undesirable situation are needed; increased uptake of telehealth PA programs and virtual exercise classes to promote PA participation might be one promising approach along with vaccination of pwCF and their close contacts.

## 1. Introduction

Severe acute respiratory syndrome–coronavirus-2 (SARS-CoV-2) became apparent at the end of 2019 and continuously causes a global pandemic affecting the health and well-being of the general population and those with chronic respiratory disease [1, 2]. According to the Centers for Disease Control and Prevention [3], people with chronic respiratory diseases such as cystic fibrosis (CF) with and without lung transplantation (LTX) are at a higher risk for severe coronavirus-disease 2019 (COVID-19); however, the clinical course and recovery of people with CF (pwCF) after COVID-19 and the potential protective role of CF transmembrane conductance regulator (CFTR) therapy remains to be investigated [4].

Reports from the European Cystic Fibrosis Society Patient Registry and Cystic Fibrosis Registry Global Data Harmonization Group aiming to characterize SARS-CoV-2 infections among pwCF show an overall mild course of COVID-19 [5–8]. LTX recipients and those with pre-existing advanced CF lung disease (ACFLD) and other extra-pulmonary comorbidities seem to be at higher risk for severe COVID-19; even fatal outcomes were reported in a few cases [5, 7, 8]. Interestingly, the incidence of SARS-CoV-2 in pwCF was reported to be higher compared to an age-matched general population [7].

In many countries, people have been advised to strictly limit social contacts and self-isolation has been recommended for pwCF to minimize the risk of SARS-CoV-2 infection. Public restrictions (e.g., closed training facilities and physiotherapy practices, omission of supervised therapy) are likely to have important implications on individuals’ therapy regimens, adherence to treatment, and mental health of pwCF [1, 9]. Our Group recently reported lower physical activity levels in a substantial proportion of adults with CF during the first wave of the pandemic including a 6-week nationwide lockdown in Switzerland [10], supported by observational data of pwCF living in the UK [9], and in Greece [11].

While most of the studies to date have reported on the impact of short periods of shielding (i.e., 3 weeks to 4 months) on treatment adherence in pwCF [1, 9–11], the long-term consequences are yet to be determined. The aim of our follow-up survey–covering the pandemic for over one year (from March 2020 to April 2021)–was to assess the continuous impact of SARS-CoV-2 on individuals’ therapy regimes, perceived health status and physical activity behavior.

## 2. Materials and methods

This is a follow-up cross-sectional study of our previous survey conducted during the first wave of the SARS-CoV-2 pandemic of pwCF living in Switzerland [10]. The self-administered online questionnaire was programmed in REDCap (Research Electronic Data Capture, Vanderbilt University, USA) and contained questions on demographics and medical characteristics, current therapies, SARS-CoV-2 vaccination status, and individuals’ perceptions towards various medical and non-medical aspects related to the SARS-CoV-2 pandemic. In addition, we assessed self-reported health status with the “feeling thermometer”, a visual analogue scale (VAS) with marked intervals from 0 (worst imaginable health state) to 100 (best imaginable health state). The feeling thermometer is part of the EuroQoL quality of life questionnaire (EQ-5D-5L) [12], a valid and frequently applied health outcome instrument in CF lung disease including lung transplant recipients [13–15].

The online questionnaire was pilot tested by three individuals with CF who also provided feedback and input on the first survey [10]. The questionnaire was made available in the three main national languages in Switzerland (French, German, Italian). The original study questionnaires were translated into English (S1 File). According to the latest European Cystic Fibrosis Society Patient Registry Report [16], 539 adults with CF live in Switzerland and belong to the pool of potentially eligibly survey participants.

We classified the severity of COVID-19 into the following stages: Stage I–- mild symptoms (i.e. dry cough, fatigue, headache); Stage II–moderate to severe symptoms (i.e., dyspnea, hypoxia); Stage III–critical symptoms (i.e., acute respiratory distress syndrome, cardiac failure); Stage IV–death [17].

On March 2^nd^, 2021, one day after easing public restrictions following the second wave of the pandemic in Switzerland (e.g., opening of retail shops, private outdoor meetings with maximal 15 persons, young people <20 years attending sports and cultural events), the online link to the REDCap questionnaire was distributed via email to all current members of Cystic Fibrosis Switzerland. In addition, the link was made available on the official Facebook^®^ page of Cystic Fibrosis Switzerland and shared in WhatsApp^®^ groups by pwCF. The Study Team additionally shared the link with the LTX and CF Center in Lausanne, and other centers caring for pwCF across Switzerland. A second survey invitation was sent as a reminder on March 16^th^, 2021. The surveys included questions covering the entire SARS-CoV-2 pandemic period from March 2020 to April 2021.

### 2.1 Ethics statement

This study does not fall under the scope of the Human Research Act. Research projects with anonymously collected or anonymous health-related personal data are exempt from requirement for approval. We paid special attention to the anonymity of survey participants, e.g., by using broad categories for age (i.e., 18–24 years, 25–39 years, 40–60 years, and >60 years) and time of LTX (i.e., < 1 year, 1–5 years, >5–10 years, > 10 years). Written consent for participation in this survey was systematically collected at the start of the online questionnaire.

### 2.2 Statistical methods

Descriptive characteristics are summarized as n (%) or median (interquartile range). Data cleaning and analysis was performed using R (version 4.0), and graphs constructed using ggplot2 (version 3.3) [18, 19].

## 3. Results

During the 4-week study period, 193 survey responses were received from pwCF. Overall, 127 (66%) of pwCF who participated in our first survey also participated in the follow-up survey.

### 3.1 Survey participants

Clinical and socioeconomic characteristics of pwCF are given in **Table 1**. Among 193 pwCF, 25% were LTX recipients, of which 3 (6%) reported to have chronic lung allograft dysfunction. Among 144 pwCF without LTX, 62% reported taking CFTR modulators, of which 42% received triple combination therapy (elexacaftor-ivacaftor-tezacaftor). Participants’ self-reported health status was different between lung disease categories: mild lung disease (FEV_1_ >80% predicted) median 84.5 (IQR 73.5, 92.3), moderate lung disease (FEV_1_ 40–80% predicted) 74.0 (65.0, 83.0), and advanced lung disease (FEV_1_ <40% predicted) 70.0 (54.5, 82.0), p = 0.001. Self-reported health status reported by lung transplant individuals was 80.0 (68.0, 90.0).

**Table 1:** Characteristics of pwCF participating.

Variables	All (n = 193)	Non-LTX (n = 144)	LTX (n = 49)
**Sex**			
Male	90 (47%)	69 (48%)	21 (43%)
Female	103 (53%)	75 (52%)	28 (57%)
**Age categories**			
18–24 years	39 (20%)	34 (24%)	5 (10%)
25–39 years	101 (52%)	77 (53%)	24 (49%)
40–60 years	49 (25%)	29 (20%)	20 (41%)
>60 years	4 (2%)	4 (3%)	0 (0%)
**Lung function (FEV**_**1**_ **)** *			
> 80% predicted		36 (25%)	–
40–80% predicted		84 (58%)	–
< 40% predicted		24 (17%)	–
**CFTR modulator therapy**			
IVA		3 (2%)	
IVA/LUM		7 (5%)	
IVA/TEZ		19 (13%)	
ELX/IVA/TEZ		60 (42%)	
**Years post LTX**			
< 1 year		–	1 (2%)
1–5 years		–	16 (33%)
>5–10 years		–	20 (41%)
> 10 years		–	12 (24%)
**Comorbidities**			
Heart disease	1 (1%)	1 (1%)	0 (0%)
Hypertension	24 (12%)	5 (4%)	19 (39%)
Diabetes	87 (45%)	46 (32%)	41 (84%)
Kidney disease/renal transplant	10 (5%)	0 (0%)	10 (20%)
Cancer^#^	2 (1%)	0 (0%)	2 (4%)

Data are presented as numbers (percent). CFTR, cystic fibrosis transmembrane conductance regulator; ELX, elexacaftor; FEV_1_, forced expiratory volume in 1s; IVA, ivacaftor; LUM, lumacaftor; LTX, lung transplantation; TEZ, tezacaftor.

*Lung function was only surveyed in non-transplant patients.

^#^Other than skin cancer.

### 3.2 Demographics on COVID-19 compatible symptoms, diagnostic testing, COVID-19 disease stages, and vaccination status

During the entire SARS-CoV-2 pandemic period starting in March 2020, 41 individuals with CF (21%) reported COVID-19 compatible symptoms. Among symptomatic subjects, 37 individuals underwent SARS-CoV-2 testing, 10 individuals (27%) were tested positive (**Table 2**). All experienced mild symptoms only—corresponding to COVID-19 Stage I [17], except two individuals who were hospitalized, one individual was admitted to the intensive care unit but with no invasive ventilation required. 80 of 193 pwCF (42%) reported being already vaccinated for COVID-19 (i.e., at least one dose of a SARS-CoV-2 two-doses vaccine regime), with a further 35% willing to get the vaccine; 8% reported not being willing to get vaccinated and the remaining 14% were undecided. 59% of survey respondents (n = 114) reported being informed by their CF center about the COVID-19 vaccination.

**Table 2:** COVID-19 like symptoms, diagnostic testing, disease stages, and vaccination status.

Variables	All (n = 193)	Non-LTX (n = 144)	LTX (n = 49)
COVID-19 like symptoms	41 (21%)	34 (24%)	7 (14%)
Symptomatic tested^§^	37 (97%)	30 (97%)	7 (100%)
SARS-CoV-2 positive	10 (27%)	8 (27%)	2 (29%)
COVID-19 Stage I*	8 (80%)	7 (88%)	1 (50%)
COVID-19 Stage II*	-	-	-
COVID-19 Stage III*	2 (20%)	1 (13%)	1 (50%)
Hospitalisation	2 (20%)	1 (13%)	1 (50%)
Intensive care	1 (10%)	1 (13%)	0 (-)
**Vaccination status**			
Already vaccinated^#^	80 (42%)	57 (40%)	23 (47%)
Not vaccinated but willing to	67 (35%)	49 (35%)	18 (37%)
Unwilling to be vaccinated	16 (8%)	15 (11%)	1 (2%)
Undecided	27 (14%)	20 (14%)	7 (14%)

Data are presented as numbers (percent). COVID-19, coronavirus disease 2019; SARS-CoV-2, Severe Acute Respiratory Syndrome Coronavirus 2.

* COVID-19 stages were adapted from Sidiqqi & Mehra^17^.

^#^ At least one dose of a SARS-CoV-2 two-doses vaccine regime.

^§^ Percentages are reported for those tested for SARS-CoV-2.

### 3.3 Impact of the SARS-CoV-2 pandemic on daily physical activity, airway clearance and inhalation therapy

**Table 3** summarizes changes in individuals’ daily maintenance therapy. During the pandemic, 48%, 5%, and 4% of individuals without LTX reported to undertake less physical activity, airway clearance and inhalation therapy, respectively. Most frequently reported reasons for doing less physical activity were closing of fitness centers (85%), lack of motivation (33%), lack of daily structure (21%), and cancellation of supervised therapy (19%). Among LTX recipients, 41% reported to undertake less physical activity. Among all survey respondents, 32 (17%) were able to increase their physical activity levels, of which 37% and 33% reported to undertake home-based training and outdoor activities more frequently; 22% reported an increase in routine physical activity, and another 8% started new activities.

**Table 3:** Impact of the SARS-CoV-2 pandemic on individuals’ daily physical activity, airway clearance and inhalation therapy.

Variables	All	Non-LTX
**Daily physical activity**		
Total subjects	193	144
No change in therapy	72 (37%)	53 (37%)
Less frequently	89 (46%)	69 (48%)
More frequently	32 (17%)	22 (15%)
**Daily airway clearance**		
Total subjects	153	136
No change in therapy	141 (92%)	124 (91%)
Less frequently	7 (5%)	7 (5%)
More frequently	5 (3%)	5 (4%)
**Daily inhalation therapy**		
Total subjects	170	141
No change in therapy	162 (95%)	133 (94%)
Less frequently	5 (3%)	5 (4%)
More frequently	3 (2%)	3 (2%)

Data are presented as numbers (percent). LTX, lung transplantation.

### 3.4 Impact of COVID-19 pandemic on individuals’ health-related and socioeconomic aspects

Perceptions of pwCF on health-related and socioeconomic aspects including concern of worsening of their lung disease, worries about their financial situation, job loss, and social isolation from the first survey and the follow-up survey are given in the online supplement (S2 File).

## 4. Discussion

To the authors’ knowledge, this is the first national survey among pwCF addressing health-related aspects related to the COVID-19 pandemic covering a one-year period. Overall, the clinical impact appears mild with 10 (5%) individuals being affected by COVID-19, no fatal outcome. COVID-19 pandemic related restrictions seem to have a continuous impact on individuals’ physical activity levels, a cornerstone therapy in the modern era of CF care, whereas daily airway clearance and inhalation therapy seem less affected.

In our cohort, the clinical impact of the SARS-CoV-2 pandemic was overall mild with 10 individuals reporting of COVID-19. Of those, two individuals (one LTX recipient) were hospitalized, one admitted to the intensive care unit but without need for invasive ventilation. Our data are consistent with recent reports from the Cystic Fibrosis Registry Global Data Harmonization Group and European Cystic Fibrosis Society Patient Registry reporting an overall mild course of COVID-19 in the majority of pwCF [5–8]. In the most recent report of the Cystic Fibrosis Registry Global Data Harmonization Group [8] including 181 pediatric and adult cases with SARS-CoV-2 covering a broad spectrum of CF lung disease severity including LTX recipients from 19 countries, 11 (6%) were admitted to the intensive care unit; 7 (4%) deaths were reported. Additionally, data collected within the European Cystic Fibrosis Society Patient Registry as of May 21 2021, confirms the overall mild clinical course of COVID-19 in pwCF in 1459 cases of which 229 were hospitalized, 33 required intensive care unit admission, 30 needed ventilatory support, and 17 pwCF died [20].

Our survey revealed long-term consequences of COVID-19 shielding on individuals’ physical activity behavior with 46% of pwCF reporting to undertake less physical activity during the pandemic. Reduced physical activity levels during earlier phases of the COVID-19 pandemic have also been reported in people living with chronic obstructive pulmonary disease and renal disease [21, 22]. Our data suggest unfavorable and prolonged effects of COVID-19 restrictions on a cornerstone therapy of CF care, given the fact that a similar proportion of pwCF in this survey (i.e., 45% in our previous survey covering a shorter time-period during the first phase of the pandemic) reported less daily physical activity. The beneficial effects of regular physical activity are well reported in pwCF without LTX, showing that physically active individuals–compared to less active individuals–have a slower rate of lung function decline, and reduced hospital admissions [23–25]. This is even more important in the era of modern CF therapy with a substantial proportion of pwCF using physical activity and exercise (i.e., structured physical activity) as a stand-alone therapy for airway clearance [26, 27]. It is important to note that Switzerland is among the European countries that experienced one of the highest second waves during the SARS-CoV-2 pandemic in Autumn 2020, but public restrictions were comparably modest. Nevertheless, access to fitness centers were limited for several months and was reported as the main reason for doing less physical activity by 85% of our survey participants. Moreover, many pwCF were advised to self-isolate and to limit social contacts, thus reducing abilities for being physically active. Fortunately, 32 (17%) of survey respondents were able to increase their physical activity levels during the pandemic. However, based on participants’ characteristics (e.g., age, sex, disease severity, CFTR modulator therapy, vaccination status, employment status and work percentage) we were not able to identify determinants for this positive behavioral change. Among those, about two-thirds chose to undertake home-based training or outdoor activities more frequently, while about one-fifth increased their usual physical activity routine, and the remaining started new activities. Moreover, in our first survey, five pwCF reported to self-organize informal virtual exercise groups. The group still exists, and they reported to meet daily during the weekdays for about 30 minutes to perform endurance type and strengthening exercises together (personal communication to first author). Telehealth interventions and virtual exercise classes to deliver and promote physical activity among pwCF appear feasible [28–30]; such tools could be further developed by healthcare professionals in collaboration with pwCF and upscaled offering supervised home-based exercise training to pwCF during periods when training facilities are closed. Moreover, virtual exercise groups offer an elegant opportunity for pwCF to exercise with their peers, that is usually not possible and not recommended due to risk of cross infection. Finally, a recent large scale analysis of 48’440 adults with COVID-19 revealed that people who are consistently physically inactive have a substantially higher risk for severe COVID-19 compared to people meeting physical activity guidelines (i.e., at least 150 min of moderate-intensity physical activity per week) [31]. To which extend these data could be extrapolated to pwCF remains to be determined; however, no doubt exists that regular physical activity is beneficial to the overall health of pwCF.

With very limited therapy options available to successfully treat COVID-19, preventive measures remain the key to prevent severe COVID-19. Just one year into the pandemic, several COVID-19 vaccines have already been approved by regulatory authorities, e.g., in the European Union, the United Kingdom, and the United States of America (USA). Although vaccines play a major role as a way out of the pandemic, willingness to receive a COVID-19 vaccination varies among people. Governments and health officials generally encourage vulnerable populations to sign up for the COVID-19 vaccination including people with chronic lung diseases such as CF. Further, patient support organizations such as the Cystic Fibrosis Foundation (CFF) in the USA strongly support COVID-19 vaccine recommendations for pwCF [32]. Our survey reveals that more than three-quarters of participating pwCF are either already vaccinated against COVID-19 or willing to do so, no difference among transplanted pwCF and non-transplanted pwCF was detected. In comparison to the general population in Switzerland and Liechtenstein–by April 2 2021–6.7% had been fully vaccinated and 4.5% had received at least one dose of a two-dose vaccination regimen, respectively [33]. To date there is limited understanding of COVID-19 vaccine immunogenicity and safety in solid organ transplant recipients as this at-risk population was excluded from the initial vaccine trials. Further, a recent study by Boyarsky et al. [34] on the immunogenicity of the mRNA SARS-CoV-2 two-dose vaccine regimes among 658 solid organ transplant recipients revealed a measurable antispike antibody response in 54% of individuals, whereas 46% had no such response, suggesting that a substantial proportion are still at risk for COVID-19 [34]. Nevertheless, the COVID-19 Taskforce of the International Society for Heart and Lung Transplantation (ISHLT) endorses vaccination for transplant recipients as the benefits of receiving SARS-CoV-2 vaccination outweigh the risks; ideally, transplant recipients should be included into vaccine trials after consultation with their responsible transplant physician [35]. Furthermore, even fully vaccinated transplant recipients should continue hygiene measures until more data are available on vaccine efficacy post-transplant. In addition, the ISHLT COVID-19 Taskforce recommends vaccination of immunocompetent household contacts of transplant recipients [35].

### 4.1 Limitations

This study has several limitations. First, with 193 survey responses, the participation rate of pwCF in this second survey was lower compared to our previous survey (n = 327, 63% response rate) covering a shorter time-period following the nationwide lockdown in Switzerland. With this second survey, we were only able to reach about 36% of potentially eligible adults with CF living in Switzerland [16]. Nevertheless, the proportion of individuals with LTX was comparable to our previous survey (25%) with no differences across age categories, sex, and lung disease severity between survey respondents potentially affecting the generalizability of our findings to adult pwCF in Switzerland. We used the same information channels to distribute the survey link and the time-period of 4 weeks was identical to our first survey. It may well be that the lower interest in this follow-up survey reflects an overall weariness (“pandemic fatigue”) among pwCF in regard to COVID-19 [36]. Second, we assessed self-reported physical activity in simple categories (“more frequently”, “less frequently”, “unchanged”) to gain insights into long-term behavioral changes potentially caused with pandemic restrictions. Objectively measured physical activity would have provided additional information about the magnitude of changes, both on an individual and population-level. However, this would have required a prospective study design and longitudinal measurements of free-living physical activity over time. Thirdly, since we assessed COVID-19 test results based on reported symptoms using branching logics in our online survey, our report may underestimate the number of positive cases due to the fact that a non-negligible proportion of positive SARS-CoV-2 tests is observed among asymptomatic pwCF [5]. Finally, individuals affected by COVID-19 or with a COVID-19 case among family, friends or acquaintances are probably more likely to complete our survey. However, among pwCF, only 21% reported COVID-19 compatible symptoms during the pandemic; thus, it seems rather unlikely that we report on a selective cohort of adults with CF.

## 5. Conclusions

In summary, our study reveals unfavorable consequences of COVID-19 pandemic restrictions on physical activity of pwCF long-term; its potential impact on physical fitness and lung health has yet to be determined. Strategies to overcome this undesirable situation are needed; increased uptake of telehealth physical activity programs and virtual exercise classes to promote physical activity participation might be one promising approach.

## Supporting information

S1 File Questionnaire to assess the state of health of people with cystic fibrosis in times of the coronavirus pandemic (COVID-19). (DOCX)

S2 File Perceptions of individuals with cystic fibrosis on health-related and socioeconomic aspects during the coronavirus pandemic. Disease categories for non-transplant individuals were based on lung function, i.e., percent predicted forced expiratory volume in 1s (FEV_1_): mild CFLD (>80%), moderate CFLD (80% - 40%), and advanced CFLD (<40%). CFLD, cystic fibrosis lung disease; LTX, lung transplantation. Red bars = first survey (n = 327, 83 LTX); Green bars = follow-up survey (n = 193, 49 LTX).
(PDF)

S3 File Individual data from all participants. (XLSX)

## References

[1] Havermans T, Houben J, Vermeulen F, Boon M, Proesmans M, Lorent N, et al. The impact of the COVID-19 pandemic on the emotional well-being and home treatment of Belgian patients with cystic fibrosis, including transplanted patients and paediatric patients. J Cyst Fibros 2020;19:880–7. 10.1016/j.jcf.2020.07.022 32768311 PMC7834420

[2] Pfefferbaum B, North CS. Mental Health and the Covid-19 Pandemic. NEJM 2020;383:510–2. 10.1056/NEJMp2008017 32283003

[3] COVID-19. Centers for Disease Control and Prevention n.d. https://www.cdc.gov/coronavirus/2019-ncov/need-extra-precautions/people-with-medical-conditions.html (accessed April 6, 2021).

[4] Peckham D, McDermott MF, Savic S, Mehta A. COVID-19 meets Cystic Fibrosis: for better or worse? Genes & Immunity 2020;21:260–2. 10.1038/s41435-020-0103-y 32606316

[5] Cosgriff R, Ahern S, Bell SC, Brownlee K, Burgel P-R, Byrnes C, et al. A multinational report to characterise SARS-CoV-2 infection in people with cystic fibrosis. J Cyst Fibros 2020. 10.1016/j.jcf.2020.04.012 32376098 PMC7183287

[6] Bain R, Cosgriff R, Zampoli M, Elbert A, Burgel P-R, Carr SB, et al. Clinical characteristics of SARS-CoV-2 infection in children with cystic fibrosis: An international observational study. J Cyst Fibros 2021;20:25–30. 10.1016/j.jcf.2020.11.021 33309057 PMC7713571

[7] Naehrlich L, Orenti A, Dunlevy F, Kasmi I, Harutyunyan S, Pfleger A, et al. Incidence of SARS-CoV-2 in people with cystic fibrosis in Europe between February and June 2020. J Cyst Fibros 2021:S1569-1993(21)00099-0. 10.1016/j.jcf.2021.03.017 34016559 PMC8053246

[8] McClenaghan E, Cosgriff R, Brownlee K, Ahern S, Burgel P-R, Byrnes CA, et al. The global impact of SARS-CoV-2 in 181 people with cystic fibrosis. J Cyst Fibros 2020;19:868–71. 10.1016/j.jcf.2020.10.003 33183965 PMC7641525

[9] Westcott KA, Wilkins F, Chancellor A, Anderson A, Doe S, Echevarria C, et al. The impact of COVID-19 shielding on the wellbeing, mental health and treatment adherence of adults with cystic fibrosis. Future Healthc J 2021;8:e47–9. 10.7861/fhj.2020-0205 33791475 PMC8004337

[10] Radtke T, Haile SR, Dressel H, Benden C. Recommended shielding against COVID-19 impacts physical activity levels in adults with cystic fibrosis. J Cyst Fibros 2020;19:875–9. 10.1016/j.jcf.2020.08.013 32878732 PMC7455146

[11] Hatziagorou E, Peftoulidou P, Toulia I, Tasika E, Chrysochoou E.A, Tsanakas J. Recommended shielding against COVID-19 impacts physical activitylevels and adherence to airway clearance therapy in patients with cysticfibrosis. J Cyst Fibros 2021;20S1:30. Conference Abstract.

[12] Herdman M, Gudex C, Lloyd A, Janssen M, Kind P, Parkin D, et al. Development and preliminary testing of the new five-level version of EQ-5D (EQ-5D-5L). Qual Life Res 2011;20:1727–36. 10.1007/s11136-011-9903-x 21479777 PMC3220807

[13] Eidt-Koch D, Mittendorf T, Greiner W. Cross-sectional validity of the EQ-5D-Y as a generic health outcome instrument in children and adolescents with cystic fibrosis in Germany. BMC Pediatr 2009;9:55. 10.1186/1471-2431-9-55 19715563 PMC2753333

[14] Singer LG, Chowdhury NA, Faughnan ME, Granton J, Keshavjee S, Marras TK, et al. Effects of Recipient Age and Diagnosis on Health-related Quality-of-Life Benefit of Lung Transplantation. Am J Respir Crit Care Med 2015;192:965–73. 10.1164/rccm.201501-0126OC 26131729

[15] Goetzmann L, Seiler A, Benden C, Boehler A, Büchi S, Jenewein J, et al. Transplantation experience as a predictor for quality of life during the first 6 months after lung transplantation. Clin Transplant 2018;32:e13393. 10.1111/ctr.13393 30151868

[16] Zolin, A, Orenti, A, Naehrlich, L, van Rens, J. European Cystic Fibrosis Society Annual Report 2018. 2020: available online: https://www.ecfs.eu/sites/default/files/general-content-files/working-groups/ecfs-patient-registry/ECFSPR_Report_2018_v1.4.pdf

[17] Siddiqi HK, Mehra MR. COVID-19 illness in native and immunosuppressed states: A clinical–therapeutic staging proposal. J Heart Lung Transplant 2020;39:405–7. 10.1016/j.healun.2020.03.012 32362390 PMC7118652

[18] R Core Team. R: A language and environment for statistical computing. R Foundation for Statistical Computing. Vienna, Austria: 2020.

[19] Wickham H. ggplot: Elegant Graphics for Data Analysis. Springer-Verlag New York; 2016.

[20] COVID-CF Project in Europe European Cystic Fibrosis Society 2021. https://www.ecfs.eu/covid-cf-project-europe (accessed June 26, 2021).

[21] Hume E, Armstrong M, Manifield J, McNeillie L, Chambers F, Wakenshaw L, et al. Impact of COVID-19 shielding on physical activity and quality of life in patients with COPD. Breathe 2020;16. 10.1183/20734735.0231-2020 33447283 PMC7792782

[22] Antoun J, Brown DJ, Jones DJW, Sangala NC, Lewis RJ, Shepherd AI, et al. Understanding the Impact of Initial COVID-19 Restrictions on Physical Activity, Wellbeing and Quality of Life in Shielding Adults with End-Stage Renal Disease in the United Kingdom Dialysing at Home versus In-Centre and Their Experiences with Telemedicine. Int J Environ Res Public Health 2021;18:3144. 10.3390/ijerph18063144 33803708 PMC8002886

[23] Schneiderman JE, Wilkes DL, Atenafu EG, Nguyen T, Wells GD, Alarie N, et al. Longitudinal relationship between physical activity and lung health in patients with cystic fibrosis. Eur Respir J 2014;43:817–23. 10.1183/09031936.00055513 24176992

[24] Cox NS, Alison JA, Button BM, Wilson JW, Morton JM, Holland AE. Accumulating physical activity in at least 10-minute bouts predicts better lung function after 3-years in adults with cystic fibrosis. ERJ Open Res 2018;4. 10.1183/23120541.00095-2017 29637075 PMC5890021

[25] Elce A, Nigro E, Gelzo M, Iacotucci P, Carnovale V, Liguori R, et al. Supervised physical exercise improves clinical, anthropometric and biochemical parameters in adult cystic fibrosis patients: A 2-year evaluation. Clin Respir J 2018;12:2228–34. 10.1111/crj.12796 29601147

[26] Rowbotham NJ, Smith SJ, Davies G, Daniels T, Elliott ZC, Gathercole K, et al. Can exercise replace airway clearance techniques in cystic fibrosis? A survey of patients and healthcare professionals. J Cyst Fibros 2020;19:e19–e24. 10.1016/j.jcf.2019.10.026 31740105

[27] Ward N, Stiller K, Holland AE, Australian Cystic Fibrosis Exercise Survey group. Exercise is commonly used as a substitute for traditional airway clearance techniques by adults with cystic fibrosis in Australia: a survey. J Physiother 2019;65:43–50. 10.1016/j.jphys.2018.11.006 30559063

[28] Chen JJ, Cooper DM, Haddad F, Sladkey A, Nussbaum E, Radom-Aizik S. Tele-Exercise as a Promising Tool to Promote Exercise in Children With Cystic Fibrosis. Front Public Health 2018;6. 10.3389/fpubh.2018.00269 30324099 PMC6172297

[29] Tomlinson OW, Shelley J, Trott J, Bowhay B, Chauhan R, Sheldon CD. The feasibility of online video calling to engage patients with cystic fibrosis in exercise training. J Telemed Telecare 2020;26:356–64. 10.1177/1357633X19828630 30803323

[30] Layton AM, Irwin AM, Mihalik EC, Fleisch E, Keating CL, DiMango EA, et al. Telerehabilitation Using Fitness Application in Patients with Severe Cystic Fibrosis Awaiting Lung Transplant: A Pilot Study. International Journal of Telemedicine and Applications 2021;2021:e6641853. 10.1155/2021/6641853 33727918 PMC7935590

[31] Sallis R, Young DR, Tartof SY, Sallis JF, Sall J, Li Q, et al. Physical inactivity is associated with a higher risk for severe COVID-19 outcomes: a study in 48 440 adult patients. Br J Sports Med 2021. 10.1136/bjsports-2021-104080 33849909

[32] Cystic Fibrosis Foundation. CF Foundation Strongly Supports COVID-19 Vaccine Recommendations for People Living With Cystic Fibrosis n.d. /News/News-Archive/2021/CF-Foundation-Strongly-Supports-COVID-19-Vaccine-Recommendations-for-People-Living-With-Cystic-Fibrosis/ (accessed June 28, 2021).

[33] COVID-19 in Switzerland. Vaccinations, Switzerland and Liechtenstein n.d. https://www.covid19.admin.ch/en/vaccination/persons (accessed September 2, 2021).

[34] Boyarsky BJ, Werbel WA, Avery RK, Tobian AAR, Massie AB, Segev DL, et al. Antibody Response to 2-Dose SARS-CoV-2 mRNA Vaccine Series in Solid Organ Transplant Recipients. JAMA 2021;325:2204–6. 10.1001/jama.2021.7489 33950155 PMC8100911

[35] International Society for Heart and Lung Transplantation. SARS-CoV-2 Vaccination in Heart and Lung Transplantation. Recommendations from the ISHLT COVID-19 Task Force 2021. https://ishlt.org/ishlt/media/Documents/COVID19_Vaccine-Recommendations_3-15-2021.pdf (accessed June 15, 2021).

[36] MacIntyre CR, Nguyen P-Y, Chughtai AA, Trent M, Gerber B, Steinhofel K, et al. Mask use, risk mitigation behaviours and pandemic fatigue during the COVID-19 pandemic in five cities in Australia, the UK and USA: a cross-sectional survey. Int J Infect Dis 2021. 10.1016/j.ijid.2021.03.056. PMC7985682 33771668

