# Supplementary material for: COVID-19 pandemic restrictions continuously impact on physical activity in adults with cystic fibrosis
Source: PLoS One. 2021 Sep 23;16(9):e0257852. doi: 10.1371/journal.pone.0257852 (PMC8460042; doi:10.1371/journal.pone.0257852)
Supplement: S1 File — (DOCX) [file pone.0257852.s001.docx]

# S1 File: Supporting Information

# Questionnaire to assess the state of health of people with Cystic Fibrosis in times of the Coronavirus Pandemic

# (COVID-19)

This questionnaire was translated from the original German version.

Language: □ German □ Français □ Italiano

I agree to participate in this survey: □ Yes □ No

# General questions

1. Did you participate in our first survey in the Spring?

□ Yes □ No

2. Biological sex:

□ Female □ Male □ Not specified

3. Age

□ 18-24 years

□ 25-39 years

□ >40 years

4. Family status:

□ No partner

□ Partner usually lives outside the household

□ Partner normally lives in the same household

□ Family with children

□ Single parent

5. Do you currently live separated from your family and/or partner due to the coronavirus pandemic?

□ Yes □ No

5a. Do you feel burdened by this spatial separation?

□ Yes □ No

6. Did you revieve a lung transplant?

□ Yes □ No

6a. Timing of lung transplantation:

□ < 1 year

□ 1 - 5 years

□ > 5 -10 years

□ >10 years

6b. Do you suffer from chronic rejection of the transplanted lung?

□ Yes □ No

6c. Do you currently have stable lung function despite chronic rejection?

□ Yes □ No

7. Information regarding your lung function: My current FEV1 value is (% predicted):

□ > 80%

□ 40 – 80%

□ <40%

8. Are you currently being treated with any of the new CF medications?

□ Yes □ No

8a. Please specify drug:

□ Kalydeco^®^ (Ivacaftor)

□ Orkambi^®^ (Ivacaftor/Lumacaftor)

□ Symdeko^®^ (Tetzacaftor/Ivacaftor)

□ Trikafta^®^ (Elexacaftor/Tezacaftor/Ivacaftor)

8b. Start Trikafta^®^

□ before February 1, 2021

□ after February 1, 2021

9. Do you have one or more of the following concomitant disease/other diagnoses that may be at increased risk of COVID-19? *Multiple answers possible*

□ No

□ Heart disease

□ High blood pressure

□ Diabetes
□ Chronic kidney disease or dialysis or kidney transplantation

□ Cancer

10. How often do you usually attend the CF or transplant center for outpatient consultations?

□ Monthly

□ every 1 - 3 months

□ every 3 - 6 months

□ every 6 - 12 months

□ > 12 months

Details of education & profession

11. Are you employed?

□ Yes □ No

11a. Level of employment (percentage)? ________%

12. In the last six months, have your work circumstances changed due to the COVID 19 pandemic?

□ Yes □ No

12a. Please specify:

□ Increase in scope of work

□ Reduction in scope of work

□ Complete or partial work from home

□ Complete cessation of professional activity

□ Partial cessation of professional activity

□ Sick leave

□ Unemployment or partial unemployment

□ Termination of the employment contract

□ Non-extension of the employment contract

□ Other

12b. If "Other", please specify: _________________

13. What is your highest education?

□ University / University of Applied Sciences / College of Education / ETH
□ Federal diploma (master craftsman diploma), Federal professional certificate / Technician or technical school / Higher technical school, HTL, HWV
□ Technical secondary school / Matura school / vocational school-leaving certificate / teacher training seminar
□ Apprenticeship / vocational training
□ Compulsory school completed
□ Compulsory school not completed

14. Do you receive an invalidity insurance?

□ Yes □ No

14a. Level of invalidity insurance (percentage)?

□ Quarter pension (40-49%)

□ Half pension (50-59%)

□ Three-quarter pension (60-70%)

□ Whole pension (70-100%)

Questions about the current situation of COVID-19

15. We would like to know how you regard your health status today.

This scale is provided with numbers from 0 to 100. 100 is the best health you can imagine. 0 (zero) is the worst health you can imagine. Please use the slider below to adjust how good or bad your health is today. *Visual analogue scale: 0, Worst health; 100, Best health*

16. Have you had at least one episode (i.e., at least 3 consecutive days) of typical COVID-19 symptoms, such as fever, increased cough, increased shortness of breath, increased headache, loss of smell or taste, in the years **since the onset of the coronavirus pandemic**? □ Yes □ No

16a. If yes, please select:

□ 1 episode

□ 2 episodes

□ 3 or more episodes

16b. What symptoms did you have during this episode? *(Multiple answers possible)*

□ General symptoms, e.g., fever, fatigue, tiredness

□ Respiratory problems such as rhinitis, cough, shortness of breath

□ Gastrointestinal symptoms such as loss of appetite, nausea, diarrhea

□ Other complaints such as loss of smell or taste, irritated eyes

16c. What did you do when the first COVID-19-like symptoms appeared?

(Multiple answers possible)

□ You stayed at home

□ You have contacted your primary care physician

□ You have contacted your CF/Transplant center

□ You went to the emergency of a hospital

□ You have called 911 (144)

□ You have not done anything special

17. Have you been tested for coronavirus (SARS-CoV-2)?

□ Yes □ No

17a. Test result?

□ Positive □ Negative

18. Have you been treated with any of the following medications specifically for COVID-19?

□ Yes □ No

18a. Please select drug(s):

□ Antibiotics

□ Cortisone

□ Hydroxychloroquine/Plaquenil ^®^

□ Antiviral agents (e.g., olseltamivir/Tamiflu^®^ , ritonavir-lopinavir/Kaletra^®^, remdesivir/Veklury^®^ )

□ I do not know

19. Were you hospitalized?

1 Hospitalization □ Yes □ No

2 Hospitalization □ Yes □ No

3 Hospitalization □ Yes □ No

19a. Have you been in the intensive care unit?

1 Hospitalization □ Yes □ No

2 Hospitalization □ Yes □ No

3 Hospitalization □ Yes □ No

19b. Mechanical ventilation?

1 Hospitalization □ Yes □ No

2 Hospitalization □ Yes □ No

3 Hospitalization □ Yes □ No

20. How do you protect yourself in the current situation? *Multiple answers possible*

□ Self-isolation

□ Home office

□ Wearing a mouth guard or FFP2/3 mask, outside the house.

□ Regular hand disinfection/hand washing

□ Other

If other, please specify: _______________

21. Were you scheduled for a regular routine check-up at your CF/Transplant Center in the **last 3 months**?

□ Yes □ No

21a. Has this routine check-up been postponed or cancelled?

□ Yes □ No

21b. Who took the initiative to postpone or cancel the routine control appointment?

□ CF Team

□ Yourself

21c. Were you offered alternatives for your routine control?

□ Yes □ No

21d. What alternatives were offered to you?

□ Online consultation hour (e.g., via Skype, Zoom, etc.)

□ Consultation telephone

□ Other

If other alternatives, please specify: __________

22. Have you been informed directly by your CF/Transplant Team about COVID-19 and recommended rules of conduct?

□ Yes □ No

22a. How were you informed by your CF/Transplant Team regarding COVID-19?

□ Consultation hour

□ Phone

□ E-mail

□ Link to homepage

□ Other

If other, please specify: _______________

22b. Do you feel sufficiently informed?

□ Yes □ No

23. Have you been informed directly by your CF/Transplant Team regarding coronavirus vaccination?

□ Yes □ No

23a. How were you informed by your CF/Transplant Team regarding coronavirus vaccination?

□ Consultation hour

□ Phone

□ E-mail

□ Link to homepage

□ Other

If other, please specify: _______________

24. I have already been vaccinated against coronavirus (SARS-CoV-2)?

□ Yes □ No

25. I will get vaccinated against coronavirus (SARS-CoV-2) as soon as I get the chance.

□ Yes □ No □ I am still undecided

Your therapy in times of COVID-19

26. Has your routine airway clearance therapy changed under pandemic conditions?

□ Yes □ No □ Not applicable

26a. Are you doing more or airway clearance therapy?

□ More □ Less □ Unchanged

26b. In case you are doing less airway clearance therapy. What are the reasons for it? *Multiple answers possible*

□ Lack of motivation
□ Lack of daily structure

□ Supervised therapy is currently cancelled (e.g., physiotherapy)

□ Other

If other, please specify: ___________

27. Has your routine daily inhalation therapy changed under pandemic conditions?

□ Yes □ No □ Not applicable

27a. Do you do more or less inhalation therapy?

□ More □ Less □ Unchanged

27b. In case you are doing less inhalation therapy. What are the reasons for it?

□ Lack of motivation
□ Lack of daily structure

□ Other

If other, please specify: ___________

28. Did your routine physical activity and exercise behaviour change under pandemic conditions?

□ Yes □ No

28a. Do you do more or less exercise and sports?

□ More □ Less □ Unchanged

28b. Have you looked for alternatives or have you been able to increase your usual exercise and sports routine? If yes, please specify: ___________________

28c. In case you are exercising less and doing less sports. What are the reasons for it?

Multiple answers possible

□ Lack of motivation
□ Lack of daily structure

□ Supervised training is cancelled (e.g., physiotherapy, medical training therapy)

□ Training opportunities are no longer available (e.g., fitness center is closed)

□ Other

If other, please specify: __________

29. The current situation causes me concern or fear of an infection with coronavirus.

□ 0 = not at all □ 100 = very much

30. The current situation causes me concern or fear of a worsening of my lung disease.
□ 0 = not at all □ 100 = very much

31. The current situation causes me concern or fear of social isolation / loneliness.

□ 0 = not at all □ 100 = very much

32. The current situation causes me concern or fear of losing my job.

□ 0 = not at all □ 100 = very much

33. The current situation worries me financially.
□ 0 = not at all □ 100 = very much

34. The current separation from my family or my partner is a great burden for me.

□ 0 = not at all □ 100 = very much

35. In the current situation, I feel more and more solidarity from my social environment towards myself.

□ 0 = not at all □ 100 = very much

36. Have you also had positive experiences in connection with the current situation regarding coronavirus? If you wish, you are welcome to share them.

Please do not share any personal identifying information and do not name any institution.

________________________________________________________________________

________________________________________________________________________

Is there anything else you would like to tell us?

________________________________________________________________________

Please do not share any personal identifying information and do not name any institution.

Thank you very much for your participation.

*The questionnaire was programmed in REDCap (Research Electronic Data Capture) containing branching logics, i.e., questions that are conditionally shown or hidden based on certain responses (field values). These links are not shown in this questionnaire version.
