# Supplementary material for: COVID-19 pandemic restrictions continuously impact on physical activity in adults with cystic fibrosis
Source: PLoS One. 2021 Sep 23;16(9):e0257852. doi: 10.1371/journal.pone.0257852 (PMC8460042; doi:10.1371/journal.pone.0257852)

Figure S2. Perceptions of individuals with cystic fibrosis on health-related and socioeconomic aspects during the coronavirus pandemic. Disease categories for non-transplant individuals were based on lung function, i.e., percent predicted forced expiratory volume in 1s (FEV_1): mild CFLD (>80%), moderate CFLD (80% - 40%), and advanced CFLD (<40%). CFLD, cystic fibrosis lung disease; LTX, lung transplantation. Red bars = first survey (n=327, 83 LTX); Green bars = follow-up survey (n=193, 49 LTX).

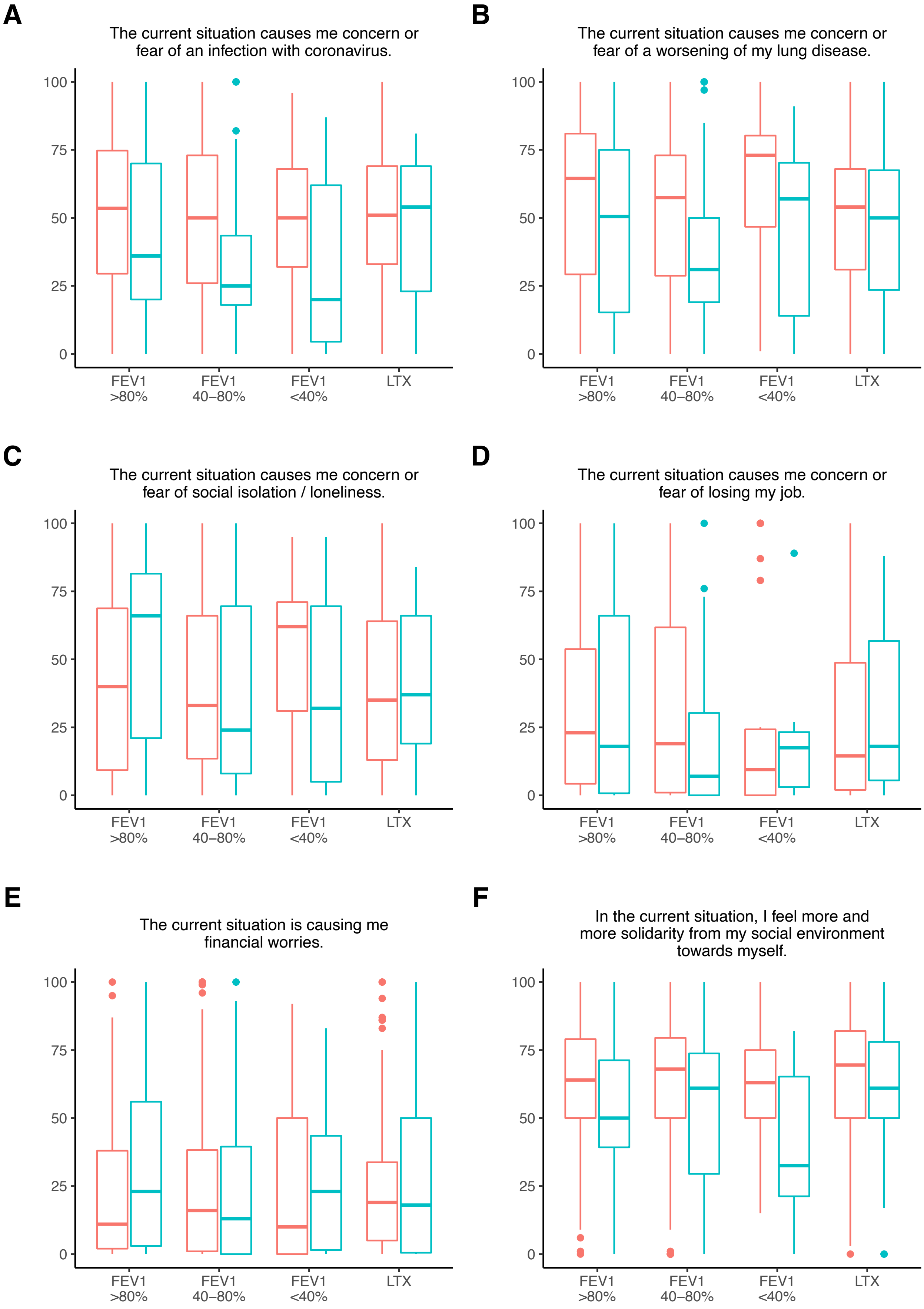

Supplement: S2 File — Disease categories for non-transplant individuals were based on lung function, i.e., percent predicted forced expiratory volume in 1s (FEV1): mild CFLD (>80%), moderate CFLD (80% - 40%), and advanced CFLD (<40%). CFLD, cystic fibrosis lung disease; LTX, lung transplantation. Red bars = first survey (n = 327, 83 LTX); Green bars = follow-up survey (n = 193, 49 LTX). (PDF) [file pone.0257852.s002.pdf]
