# Supplementary material for: COVID-19 pandemic restrictions continuously impact on physical activity in adults with cystic fibrosis
Source: PLoS One. 2021 Sep 23;16(9):e0257852. doi: 10.1371/journal.pone.0257852 (PMC8460042; doi:10.1371/journal.pone.0257852)
Supplement: Attachment — Submitted filename: Response to Reviewers.docx [file pone.0257852.s004.docx]

**PONE-D-21-21163**

**COVID-19 pandemic restrictions have long-term impact on physical activity in adults with cystic fibrosis**

Reviewers' comments:

**Reviewer #1:** In this paper the authors present the second online survey to explore the impact of pandemic restrictions to combat COVID-19 on pwCF therapeutic regimens and physical well-being. The authors also included CF patients with lung transplantation. The results confirm that the pandemic negatively affects all patients as a significant number of them stop exercising, and the others are training autonomous and uncontrolled activities, therefore the long-term consequences on the physical and lung health of these patients are not foreseeable.
The work is interesting and confirms the data reported in a previous work by the same authors, and it is important to reiterate that physical activity is a fundamental practice for the anthropometric, clinical, and metabolic improvement of pwCF regardless of the pandemic, as reported in the work of Elce et al, published in Clin Respir J. 2018; 12 2228–2234.

Response. We thank the Reviewer for acknowledging the clinical relevance of our work and also for suggesting an additional reference. With interest, we read the single-center observational study by Elce et al. on the effects of regular physical activity on overall health in people with CF.

Authors should cite this work at the following points in the manuscript:
- page 3, line 67 in addition to the other citations;

Response: This sentence focuses on the impact of public restrictions on individuals’ therapy regimes, adherence to treatment and mental health. As the suggested reference would not fit well in this context, we opted to not cite this reference here.

- page 13, line 209, change "regular" with "supervised" and "activity" with "exercise";

Response: Throughout the manuscript, we use the term “physical activity” that includes both sporadic activities and structured exercise. Additionally, our survey questions focused on “physical activity” rather than structured exercise alone. Thus, we would prefer to keep the term “physical activity” in this sentence, consistent with the terminology used throughout the manuscript.

Moreover, in this sentence, we cited two observational studies. Replacing “regular” with “supervised” in this sentence would not be appropriate as those studies were not supervised interventions.

- page 13, line 211, add after admission, "and improves clinical, anthropometric and metabolic parameters;

Response: As suggested by the Reviewer, we referenced the paper by Elce et al. in the context of reduced ppFEV_1_ decline in physically active *versus* physically inactive individuals. The sentence now reads: “The beneficial effects of regular physical activity are well reported in pwCF without LTX, showing that physically active individuals – compared to less active individuals – have a slower rate of lung function decline, and reduced hospital admissions [19–21].”

- page 16, line 283, after "over time".

Response: The study by Elce et al. compares adults with CF that regularly perform physical exercise over 3 years to a matched control group of adults with CF without regular physical exercise. Structured exercise (e.g., treadmill running, jogging, swimming, running etc) was qualitatively assessed by means of weekly sessions (Table 1 in original paper). This is different to the measurement of free-living physical activity that also includes sporadic activity. Moreover, in the LIMITATIONS section, we highlight the objective measurement of physically activity that was not performed in the study by Elce et al. For these reasons, we opted not to cite this study here.

**Reviewer #2:** This is an interesting follow up study regarding physical activity levels and maintenance therapy adherence in CF patients during the COVID-19 pandemic. Even though it received fewer participants’ responses than the original study it was still representative of the population and this limitation was well noted on the manuscript. The authors provided an adequate review of the literature and the manuscript is well organized.
However I would suggest some reviews:

Response: We thank the Reviewer for acknowledging the quality of our work.

1- In the Introduction section the authors list as one of their objectives to assess the long-term impact of SARS-CoV-2 on individuals’ perceived health status. However, this is not approached in the results or the conclusions. The questionnaire has one question regarding perceived health status (question 15: “We would like to know how you regard your health status today”), but answers are not described in the study. It would be interesting to include this data, and to evaluate whether the change in physical activity were associated with perceived health status.

Response: The Reviewer is completely right. In the revised manuscript, we added the data for self-reported health status, see METHODS and RESULTS section, it now reads as follows.

METHODS: (page 4). In addition, we assessed self-reported health status with the “feeling thermometer”, a visual analogue scale (VAS) with marked intervals from 0 (worst imaginable health state) to 100 (best imaginable health state). The feeling thermometer is part of the EuroQoL quality of life questionnaire (EQ-5D-5L) [13], a valid and frequently applied health outcome instrument in CF lung disease including lung transplant recipients [14–16]”.

RESULTS: (page 6): “Participants’ self-reported health status was different between lung disease categories: mild lung disease (FEV_1_ >80% predicted) median 84.5 (IQR 73.5, 92.3), moderate lung disease (FEV_1_ 40-80% predicted) 74.0 (65.0, 83.0), and advanced lung disease (FEV_1_ <40% predicted) 70.0 (54.5, 82.0), p=0.001. Self-reported health status reported by lung transplant individuals was 80.0 (68.0, 90.0)”.

Additionally, as suggested, we investigated whether changes in physical activity were associated with self-reported health status. In the graph below, self-reported health status is displayed for individuals who reported unchanged, lower and higher physical activity according to disease severity status and LTX. We display the data for different disease states as we found in our previous survey (Radtke et al. JCF 2020) that self-reported health status is negatively associated with disease severity (i.e., ppFEV_1_ categories).

One can notice a large variability in self-reported health status (see right panel, n=193) within the study population; even in those with mild disease (left panel). In some of the categories, the sample size is very small (e.g., n=3-5 individuals). Therefore, the data need to be interpreted with caution and – for these reasons – we did not further investigate associations with more complex statistical models (i.e., regression models).

2- It would also be interesting to investigate whether vaccination status was associated to higher physical activity.

Response: Thank you for this interesting suggestion. We investigated associations between vaccination status and physical activity categories (see Table below). There was no indication for an association between vaccination status and physical activity category (p=0.32), and the proportion of people reporting an increase in physical activity was not different between vaccinated and non-vaccinated individuals (18.8% *versus* 14.5%).

|  |  |  | Vaccination status | |
| --- | --- | --- | --- | --- |
|  |  | Overall  (n=193) | Already vaccinated  (n=80) | Not vaccinated (n=110) |
| Physical activity | Less | 89 (46.1) | 40 (50.0) | 48 (43.6) |
|  | Unchanged | 73 (37.3) | 25 (31.2) | 46 (41.8) |
|  | More | 32 (16.6) | 15 (18.8) | 16 (14.5) |

Data are given in numbers (%).

3- In Supplement 2, the colors in the figures are lacking captions.

Response: We created a new file (S2_Supporting Information) including the figure legend.

4- In section 3.3 the reduction of airway clearance and inhalation therapy is noted twice (in line 159 and 166).

Response: Thank you. We removed the sentence duplicating the information on changes in airway clearance and inhalation therapy.

**Reviewer #3:** Thank you for giving me the opportunity to read this very interesting survey by Radtke et al. Overall, the authors should be acknowledged for what it a well-conducted survey and a clear and concise reporting of these valuable results. The COVID-19 pandemic have raised very important questions for the CF community of patients and caregivers, and this survey provides relevant results and appropriate messages.

Response: Thank you for acknowledging the importance of our survey and the quality of our work.

My main concern is about the terminology employed by the authors concerning the "long-term" effect of the pandemic restrictions. I am not fully comfortable with this wording of "long term consequences" employed by the authors. I fully agree with the interest of this one-year survey but, to my opinion, the pandemic restrictions are still widely applied in many countries or have been relaxed in the very recent past (after April 2021 in France for example). I am not fully aware of the precise situation in Switzerland but quoting the authors stated in their Methods section, restrictions "were eased on March 1st 2021".
Under these circomstances, it appears that this survey may not be investigating the long-term consequences of the primary pandemic restrictions but is rather exploring the "continuous" restrictions that have been applied during the last year. The wording "long-term" could be referring to previous restrictions abandoned for a long time but that still have consequences at the time of writing, which is obviously not the case here. Those consequences are still particularly relevant today, and as much important for the CF community but the terminology emmployed could be rethought.

Response: Thank you for your careful thoughts. We fully agree that the pandemic restrictions were present for a long time and relaxed in-between (e.g., opening of fitness centres, opportunity for pwCF to perform supervised physiotherapy etc.). Therefore, we adapted our manuscript title to better reflect the situation of continuous restrictions and its impact on physical activity in pwCF. The revised title now reads: “COVID-19 pandemic restrictions continuously impact on physical activity in adults with cystic fibrosis”. Further, we adapted the terminology in the DISCUSSION section and replaced the term “long-term” with “continuous” or “prolonged”.

My other comments are minor:

Abstract : P2, L35-38 : It seems quite unclear whether the following percentages ("37% and 33% reported to undertake home-based training and 37 outdoor activities more frequently; 22% reported an increase in routine PA, and another 8% 38 started new activities.") are related to the whole study sample or precisely the 32 pwCF that increased daily PA. One would implicitely understand the latter, but it should be precised if that is the case.

Response: Indeed, with the provided information, we were referring to people able to increase their PA levels (n=32). We added numbers to the reported percentages to provide additional clarity for the reader. It now reads: “In contrast, 32/193 (17%) pwCF were able to increase their PA levels: 12 (38%) and 11 (34%) reported undertaking home-based training and outdoor activities more frequently; 6 (19%) reported an increase in routine PA, and another 3 (9%) started new activities.”

Introduction: P3, L65-67: In France, the list of public restrictions proposed by the authors should have encompassed the closing of physiotherapy practices. Should it have also been the case in Switzerland, it should be noticed since many CF patients were advised to continue their physiotherapy treatments (including exercise training) at home, most of them without any supervision.

Response: The Reviewer is correct. The situation in France was similar to Switzerland in that regard. We added closing of physiotherapy practices to the sentence on page 3. It now reads: “Public restrictions (e.g., closed training facilities and physiotherapy practices, omission of supervised therapy) are likely to have important implications on individuals’ therapy regimens, adherence to treatment, and mental health of pwCF [1,10].”

Methods: P4, L92-101: Did the authors calculated the number of potential responders for this survey ? Considering the distribution channels employed, it appears that a large amount of pwCF in Switzerland were able to access this questionnaire. This would be helpful to state the representativity of the survey results in the Results and the Limitations section.

Response: Thank you for this important remark. According to the latest ECFS Patient Registry Report published in 2020, 539 adults with CF live in Switzerland, and 193 individuals from various regions across Switzerland participated in our study. Many pwCF are members of Cystic Fibrosis Switzerland, the national patient organisation, who supported us distributing the survey. Additionally, the survey link was circulated by several specialised CF centers and one lung transplant center and made available on the Cystic Fibrosis Switzerland website and Facebook site.

We added the following information to the METHODS section on page 4: “According to the latest European Cystic Fibrosis Society Patient Registry Report 539 [17] adults with CF live in Switzerland and belong to the pool of potentially eligibly survey participants.“

We further added information on the representativeness of our study sample to the LIMITATIONS section. The paragraph now reads: “This study has several limitations. First, with 193 survey responses, the participation rate of pwCF in this second survey was lower compared to our previous survey (n=327, 63% response rate) covering a shorter time-period following the nationwide lockdown in Switzerland. With this second survey, we were only able to reach about 36% of potentially eligible adults with CF living in Switzerland [17]. Nevertheless, the proportion of individuals with LTX was comparable to our previous survey (25%) with no differences across age categories, sex, and lung disease severity between survey respondents potentially affecting the generalizability of our findings to adult pwCF in Switzerland.”

Methods: P4, L92: It is stated in the supporting information that the questionnaire was programmed in Redcap. Was Redcap used only for the reporting of each questionnaire to facilitate the statistical analysis? Or was it used by each of the respondents to report their answers ? If so, it should be stated in the Methods section since it would be a strength of the survey in terms of personal data protection. If not, the platform on which respondents completed the questionnaire should be specified here.

Response: We used REDCap to programme the survey and a survey link to distribute the REDCap survey via different information channels. We added this information to the METHODS section: “The self-administered online questionnaire was programmed in REDCap (Research Electronic Data Capture, Vanderbilt University, USA) and contained questions on demographics and medical characteristics, current therapies, SARS-CoV-2 vaccination status, and individuals’ perceptions towards various medical and non-medical aspects related to the SARS-CoV-2 pandemic.”

“On March 2^nd^, 2021, one day after easing public restrictions following the second wave of the pandemic in Switzerland (e.g., opening of retail shops, private outdoor meetings with maximal 15 persons, young people <20 years attending sports and cultural events), the online link to the REDCap questionnaire was distributed via email to all current members of Cystic Fibrosis Switzerland”.

Methods: P5, L96: "on the" is repeated twice

Response: Removed, thank you.

Results: Well reported and well-written section.

Response: Thank you.

Discussion: The discussion section is also well-written. Messages are clear and thoughtful.

Discussion: P14, L241: The authors could precise here the percentage of people fully vaccinated, or partially vaccinated in the general population in Switzerland. Vaccination coverage is still very heterogeneous between European countries today and it would be interesting to add a comparative element to support the results.

Response: We added the following information to the DISCUSSION section, as suggested (page 16). It now reads: “Our survey reveals that more than three-quarters of participating pwCF are either already vaccinated against COVID-19 or willing to do so; no difference among transplanted pwCF and non-transplanted pwCF was detected. In comparison to the general population in Switzerland and Liechtenstein – by April 2 2021 – 6.7% had been fully vaccinated and 4.5% had received at least one dose of a two-dose vaccination regimen, respectively [34].”
